# Dual-functional injectable adhesive hydrogel delivering ginger-derived doxorubicin vesicles for osteosarcoma recurrence suppression and post-resection wound healing

**DOI:** 10.3389/fbioe.2025.1609673

**Published:** 2025-06-19

**Authors:** Qiang Zhang, Yu Zhang, Hui Chen, Lei-Na Sun, Bin Zhang, Dong-Sheng Yue, Chang-Li Wang, Zhen-Fa Zhang

**Affiliations:** ^1^ Department of Lung Cancer, Tianjin Medical University Cancer Institute and Hospital, National Clinical Research Center for Cancer, Tianjin, China; ^2^ Key Laboratory of Cancer Prevention and Therapy, Tianjin, China; ^3^ Tianjin Lung Cancer Center, Tianjin, China; ^4^ Tianjin’s Clinical Research Center for Cancer, Tianjin, China; ^5^ Department of Pathology, Tianjin Medical University Cancer Institute and Hospital, Tianjin, China

**Keywords:** postoperative osteosarcoma management, ginger vesicles, adhesive hydrogel, doxorubicin, antioxidation-enhanced bone regeneration, tumor recurrence inhibition

## Abstract

**Introduction:**

Elderly osteosarcoma patients often face significant postoperative challenges, including high recurrence rates and delayed wound healing. These issues are primarily due to inadequate hemostasis, reactive oxygen species (ROS)-mediated microenvironmental inhibition, and compromised bone regeneration. This study aims to address these challenges by introducing a multifunctional adhesive hydrogel designed for synergistic therapy.

**Methods:**

The hydrogel consists of carboxymethyl chitosan methacryloyl (CMCSMA) and tannic acid (TA), which form a dynamic, crosslinked polymer network capable of rapid tissue adhesion and adaptability to moist wound environments. The hydrogel incorporates ginger vesicles (GVs) loaded with doxorubicin (DOX), offering a dual therapeutic approach. The system facilitates hemostasis through physical barrier formation and activation of coagulation factors, while GVs and DOX provide controlled release for ROS scavenging, reduction of inflammation, and targeted tumor cell elimination.

**Results:**

*In vitro* experiments demonstrated the hydrogel’s ability to efficiently remove ROS and promote osteogenic differentiation. In a rat osteosarcoma resection model, the hydrogel significantly shortened hemostasis time compared to conventional sponges, reduced tumor recurrence, and accelerated wound healing.

**Discussion:**

This study presents a multifunctional hydrogel that combines hemostasis, antioxidation, tissue repair, and recurrence prevention. The findings suggest that this integrated therapeutic approach holds substantial potential for clinical application in elderly osteosarcoma treatment, addressing critical postoperative challenges and improving patient outcomes.

## 1 Introduction

Elderly osteosarcoma patients face unique challenges, including high tumor burden, impaired tissue repair, and poor bone healing. While surgical resection remains the primary treatment for osteosarcoma (Imura et al., 2019; Iwata et al., 2014), the risks of tumor recurrence and postoperative wound complications remain significant clinical concerns (Nagano et al., 2020). Recurrence—largely driven by residual tumor cells left behind after surgery—is a major cause of mortality (Hiller et al., 2018). Although conventional chemotherapy is commonly used to target these cells, its efficacy is often limited by poor delivery efficiency and non-specific cytotoxicity (Fearon et al., 2013). Additionally, chronic wounds at the surgical site can lead to prolonged inflammation, which not only increases the likelihood of tumor recurrence but also hinders bone regeneration (Newman et al., 2021; Ivanovski et al., 2011; Ag et al., 2017). Therefore, there is a pressing need for a postoperative adjuvant therapy that can both eliminate residual tumor cells and improve the local microenvironment to enhance wound healing and bone repair.

In recent years, natural nanovesicles have garnered significant interest in drug delivery due to their ability to improve the solubility, stability, and targeted transport of therapeutic agents (Man et al., 2021; Kusuzaki et al., 2017; Bruno et al., 2022; Zhang R. et al., 2023; Rajaram et al., 2024; Vader et al., 2016). Among them, extracellular vesicles and biomimetic vesicles show particular promise for delivering phytochemicals with poor water solubility and limited pharmacokinetics, especially in cancer therapy (Sharma et al., 2025; Charoenviriyakul et al., 2018; Kulikova et al., 2018), and bone regeneration (Lei et al., 2021). Ginger-derived vesicles (GVs), nanoscale structures isolated from ginger, offer a cost-effective, easily accessible, and biocompatible platform with low immunogenicity (Liu et al., 2025). Their unique composition enables efficient encapsulation and targeted delivery of therapeutic agents to diseased tissues, such as tumors, enhancing drug efficacy. Notably, GVs naturally contain bioactive compounds like curcumin, gingerol, and shogaol, which possess strong antioxidant properties that reduce oxidative stress and protect cells from free radical damage (Casula et al., 2022). These antioxidant activities are especially relevant to elderly osteosarcoma patients, who often suffer from elevated oxidative stress and impaired redox homeostasis that hinder healing and accelerate cellular senescence. Furthermore, the protein-, lipid-, and carbohydrate-rich surfaces of GVs can be chemically or genetically modified to expand their functional potential. Beyond their role in oncology, GVs have demonstrated regenerative properties, promoting wound healing and osteogenesis. They also exert pro-angiogenic effects that may counteract age-associated microvascular insufficiency, thus improving nutrient delivery and tissue perfusion in elderly patients. These multifunctional capabilities position GVs as a powerful plant-derived nanocarrier for both cancer therapy and tissue repair, particularly in osteosarcoma management.

To further enhance therapeutic outcomes, various advanced strategies—such as photothermal therapy (PTT), photodynamic therapy (PDT), radiosensitizers, immunomodulators, and chemotherapeutics—have been integrated into hydrogel matrices for combination therapy (Chen et al., 2021; Li et al., 2023; Lima-Sousa et al., 2023). However, these approaches often trigger local stress responses, including chronic inflammation (Li et al., 2020; Hu et al., 2024), which can impair wound healing and hinder bone regeneration after surgery. To address this, combining chemotherapy with anti-inflammatory therapy has emerged as a promising postoperative strategy, though further preclinical validation is required. Recent advances have led to the development of multifunctional platforms that integrate tumor suppression with anti-inflammatory effects to prevent recurrence and promote tissue repair. For example, doxorubicin (DOX)-loaded hydrogels have demonstrated potential in chemo-photothermal or chemo-photodynamic synergistic therapies while simultaneously reducing inflammation, thereby enhancing the postoperative microenvironment (Sabino et al., 2021; Zhuang et al., 2022).

However, conventional drug delivery systems are often limited by rapid clearance and lack of sustained release, reducing their ability to continuously modulate inflammation and control recurrence. Additionally, they typically fail to provide rapid hemostasis or enhance wound healing, further complicating postoperative recovery. These limitations underscore the need for innovative biomaterial-based solutions (Tezcan et al., 2024; Wen et al., 2022; Zhang et al., 2017). In this regard, adhesive hydrogels have shown significant promise in drug delivery, wound healing, and tissue regeneration (Liang et al., 2019; Turabee et al., 2019). Their high water content, excellent gas permeability, and biomimetic extracellular matrix (ECM)-like structure enable efficient drug loading (Fu et al., 2021), sustained release (Wang et al., 2024), and robust tissue adhesion (Fu et al., 2021), making them highly suitable for biomedical applications. When engineered with composite or dual-network structures, they can effectively fill tissue defects, promote hemostasis following osteosarcoma resection, and facilitate tissue regeneration.

Adhesive hydrogels also serve as effective exosome carriers capable of reducing reactive oxygen species (ROS), enabling prolonged drug release, and enhancing both wound healing and bone repair. This multifunctional approach minimizes systemic toxicity while supporting localized tissue regeneration. In particular, hydrogels crosslinked with carboxymethyl chitosan methacryloyl (CMCSMA) and tannic acid (TA) exhibit strong tissue adhesion, hemostatic effects, and antioxidant properties. These features enhance drug stability and allow precise delivery of therapeutic agents such as GVs, further amplifying their therapeutic value. Together, these properties underscore the potential of CMCSMA-TA-based hydrogels as next-generation platforms for postoperative osteosarcoma therapy.

**FIGURE 1:**
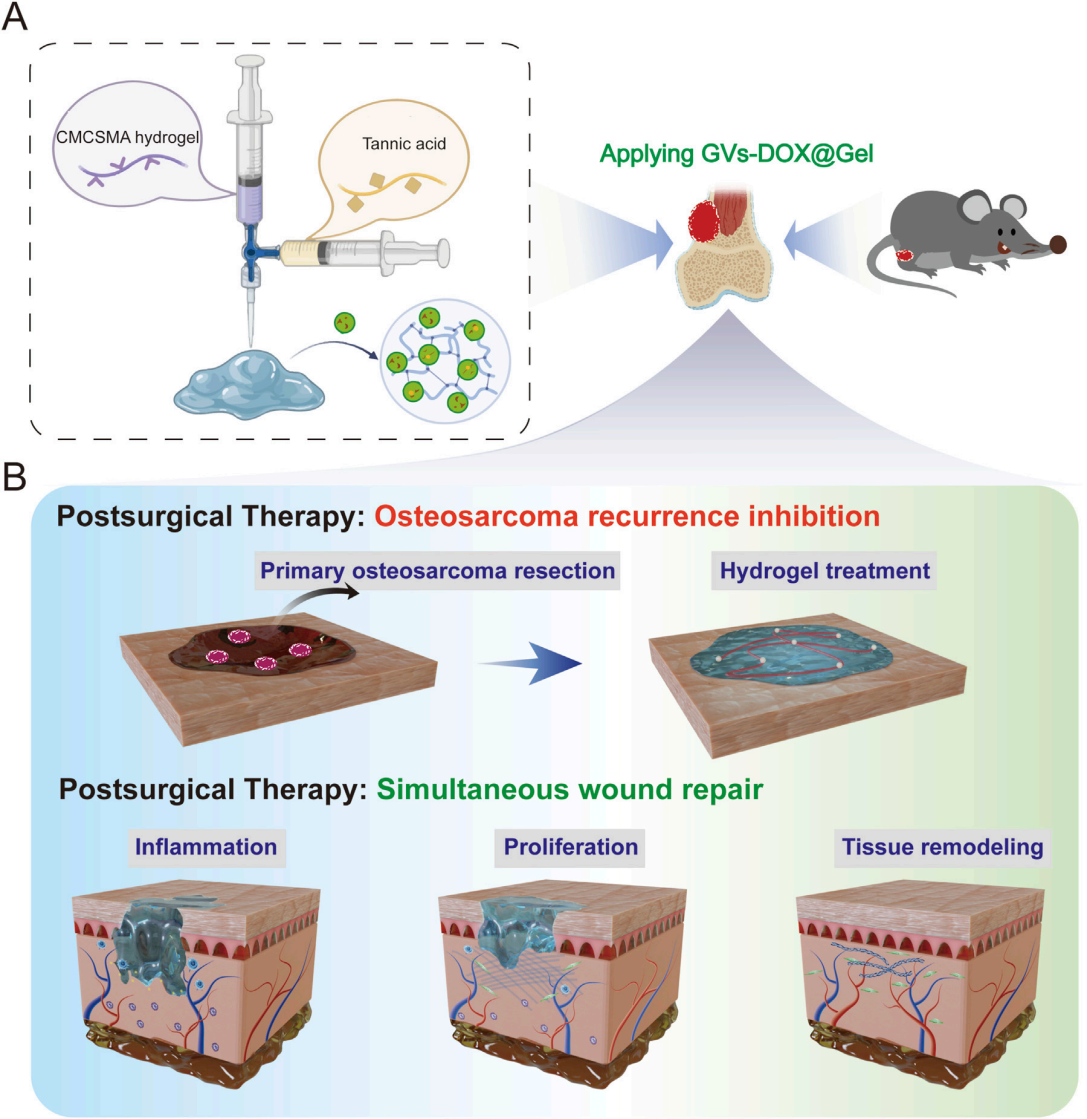
Schematic illustration of **(A)** CMCSMA-TA hydrogel loaded with DOX@ ginger vesicles and **(B)** Postoperative treatment: preventing tumor recurrence and promoting wound healing.

Building on these principles, this study presents a dynamically crosslinked natural polymer hydrogel composed of CMCSMA and TA, incorporating GVs loaded with DOX into a GelMA matrix (GVs-DOX@Gel) for targeted tumor therapy (Figure 1). Made from biocompatible materials, GVs-DOX@Gel exhibits excellent injectability, hemostatic properties, and overall biocompatibility, along with strong antitumor efficacy. The engineered GVs-DOX nanoparticles enhance DOX’s therapeutic effect, enabling efficient tumor cell elimination. Sustained hydrogel retention at the tumor site ensures prolonged drug release, maintaining high local DOX concentrations while modulating the tumor microenvironment by reducing inflammation and enhancing antitumor responses. Beyond tumor suppression, the system also alleviates oxidative stress, promotes fibroblast recruitment and proliferation, and improves the wound microenvironment, thereby accelerating postoperative healing. Overall, this multifunctional biomaterial offers a promising platform for combined tumor therapy and tissue regeneration in postoperative applications, addressing key challenges in osteosarcoma treatment.

## 2 Materials and methods

### 2.1 Materials

Hydrogen peroxide (H_2_O_2_, 30%) and DOX were purchased from Aladdin Chemistry (Shanghai) Co., Ltd. Phosphate-buffered saline (PBS) was obtained from Biosharp Biotechnology. Fresh ginger was sourced from a local market in Tianjin. The CCK-8 kit, sucrose, Calcein-AM/PI, DAPI staining solution, and Nile red staining solution were provided by Sangon Biotech (Shanghai). Crystal violet staining solution (0.1%) was supplied by Solarbio Life Sciences (Beijing, China). Dichlorodihydrofluorescein diacetate (DCFH-DA) and trypsin were purchased from Shanghai Shaoxin Biotechnology Co., Ltd. Fetal bovine serum (FBS), DMEM/high glucose medium, and RPMI-1640 medium were obtained from Thermo Fisher Scientific (China) Co., Ltd. Alizarin Red S (ARS) and alkaline phosphatase (ALP) staining kits were purchased from Cyagen (China). All other chemicals used were of analytical grade.

### 2.2 Preparation of ginger vesicles

Fresh ginger was thoroughly washed with ultrapure water, dried, weighed, and cut into small pieces. The pieces were homogenized in 0.1 M PBS for 3 min using a homogenizer. The resulting homogenate was filtered, and the filtrate was collected and centrifuged at 4,000 × g for 60 min at 4°C. The supernatant was collected and subjected to successive centrifugation at 8,000 × g and 12,000 × g for 60 min each at 4°C. The final supernatant was then centrifuged at 16,000 × g for 60 min at 4°C, and the resulting precipitate was collected and resuspended in PBS.

To further purify the vesicles, 10% PEG6000 was added to the suspension, followed by centrifugation at 16,000 × g for 60 min at 4°C. The precipitate was collected and resuspended in PBS. A 25% sucrose solution was then added, and the mixture was centrifuged at 10,000 × g for 30 min at 4°C. The final precipitate was resuspended in PBS and filtered through a 0.45 μm membrane to obtain the purified GV solution.

### 2.3 Preparation and characterization of DOX loaded GVs

GVs and DOX were mixed at a mass ratio of 1:2 and sonicated using an ultrasonic cell disruptor (JY92-IIDN, Ningbo Scientz Biotechnology Co., Ltd., China) at 1% amplitude for 10 cycles (2 s on/2 s off). Unloaded DOX was removed by ultrafiltration, and the GVs-loaded DOX was incubated at 37°C for 1 h. Zeta potential measurements before and after DOX loading were performed using a Nano-ZS 90 Nanosizer (Malvern Instruments Ltd., United Kingdom). Transmission electron microscopy (TEM) imaging was conducted with a JEM210 microscope (Japan) at 200 kV. A 5 μL droplet of the ginger vesicle solution was placed on a carbon-coated copper grid and air-dried at room temperature.

For GVs-DOX colocalization analysis, purified GVs (1 × 10^10^ particles/mL, quantified by nanoparticle tracking analysis) were labeled with PKH67 green fluorescent dye (2 μM in Diluent C, Sigma-Aldrich) via hydrophobic insertion at 25°C for 20 min under gentle agitation. Unbound dye was removed by ultracentrifugation (100,000×g, 4°C, 2 h) through a 30% sucrose cushion. DOX was fluorescently labeled with Cy5.5 NHS ester (Lumiprobe; excitation/emission: 675/694 nm) at a 1:5 M ratio via amine coupling. Excess dye was removed using Zeba™ Spin Desalting Columns (7K MWCO, Thermo Fisher). Fluorescent imaging was performed using a Leica TCS SP8 STED 3X confocal microscope.

### 2.4 Synthesis and characterization of DOX-GVs loaded CMCSMA/TA hydrogel

The synthesis of CMCSMA was performed as described in previous studies and detailed in the Supplementary Material (Bucciarelli et al., 2024; Schuurmans et al., 2021). For hydrogel formation, freshly prepared solutions were used: 10% (w/v) CMCSMA, 5% (w/v) TA, 0.5 M HCl, and 4% (w/v) NaHCO_3_. Specifically, 1 mL of 10% CMCSMA solution was mixed with 225 μL of 5% TA solution and 50 μL of 0.5 M HCl. After thorough mixing, 100 μL of 4% NaHCO_3_ was added, and the solution was immediately shaken for approximately 1 min. Gelation was achieved through photocrosslinking for 5 min.

To characterize the hydrogel, samples were flash-frozen in liquid nitrogen and lyophilized. The surface morphology of the resulting hydrogels and nanoparticles was examined using scanning electron microscopy (SEM, Hitachi Regulus 8230). Fourier-transform infrared spectroscopy (FTIR, Thermo Fisher Nicolet 6700) was conducted in the 500–4,000 cm^−1^ range to analyze chemical structures. The dynamic rheological properties, including storage modulus and loss modulus, were evaluated using a rotational rheometer (Anton Paar, Graz, Austria) as a function of temperature. To assess drug release kinetics, hydrogels were immersed in 1 mL of PBS (pH 7.4) and incubated at 37°C in a humid environment. At predetermined time intervals, the DOX concentration in the release medium was measured via ultraviolet-visible (UV-Vis) spectrophotometry. The entire PBS volume was replaced with fresh buffer each time to maintain sink conditions throughout the release study.

### 2.5 Intraoperative hemostasis evalution of CMCSMA/TA hydrogel

The hemostatic efficacy of the CMCSMA/TA hydrogel for deep, incompressible wounds was evaluated using a liver incision model, as described in previous studies (Jiang et al., 2024). In this model, a 0.5 cm deep incision was made in the liver using a scalpel to induce bleeding. Then, 100 µL of preformed hydrogel was applied directly to the wound site. Hemostasis time and total blood loss were recorded to assess efficacy. A no-treatment group served as the control.

### 2.6 Anti-tumor effects of DOX-GVs *in vitro*

To evaluate cellular uptake, Cy5-labeled DOX was loaded into GVs (Cy5-DOX@GVs) and visualized using fluorescence microscopy. MNNG/HOS and bone marrow-derived mesenchymal stem cells (BM-MSCs) were seeded in 6-well plates at a density of 5 × 10^5^ cells per well, incubated overnight, and treated with Cy5-DOX@GVs for 4 h. Cells were then washed, stained with 1 μM DAPI for 30 min, and imaged using a fluorescence microscope (Nikon, Japan). For cytotoxicity assessment, 2 × 10^4^ MNNG/HOS cells were seeded into 48-well plates with 1 mL of complete DMEM. After 12 h of incubation, 50 μL of various hydrogels or PBS (control) were added. Following a 24-h treatment, live/dead cell staining was performed using Calcein-AM and PI. To assess cell migration, a Transwell assay was conducted. A total of 2 × 10^4^ MNNG/HOS cells in 100 μL of serum-free DMEM were seeded into the upper chamber of 24-well Transwell inserts (8 μm pore size, BD Biosciences). The lower chambers were filled with DMEM containing 20% FBS as a chemoattractant. After 24 h of incubation, cells were fixed with 4% paraformaldehyde for 15 min, stained with 0.1% crystal violet for 30 min, and imaged under a light microscope. Migrated cells were quantified by counting the average number per field.

### 2.7 Pro-tissue repair effects of DOX-GVs *in vitro*

To assess the ROS scavenging ability and corresponding cytoprotective effects of the hydrogels, BM-MSCs cells were seeded at a density of 1 × 10^5^ cells per well in 48-well plates with 1 mL of complete DMEM. After 12 h of incubation, the medium was replaced with 1 mL of DMEM containing 1 mM H_2_O_2_ and 100 μL of the respective hydrogels or PBS (control). Following 2 h of incubation, the hydrogels were removed, and the wells were gently rinsed with PBS. Cells were then stained with DCFH-DA and Hoechst 33,342, and observed under a fluorescence microscope to evaluate intracellular ROS levels. To assess cell proliferation, BM-MSCs (1 × 10^4^ cells per well) were seeded in 96-well plates pretreated with different hydrogel formulations and cultured for 1, 3, and 7 days. Cell viability was measured using a CCK-8 assay, with absorbance recorded at 450 nm using a microplate reader. For quantitative analysis of anti-inflammatory, anti-senescence, and osteogenic gene expression, BM-MSCs were seeded at 2 × 10^5^ cells per well in 6-well plates and incubated overnight with or without hydrogels. The following day, cells were treated with or without 0.5 μg/mL lipopolysaccharide (LPS) for 24 h. After stimulation, total RNA was extracted, reverse transcribed, and subjected to quantitative real-time PCR (RT-PCR) using a SYBR Green Master Mix Kit. Primer sequences are provided in Supplementary Table S1.

### 2.8 *In Vitro* studies of osteoclastogenesis

Mouse bone marrow-derived macrophages (BMDMs) were isolated from the femurs of female mice (6–8 weeks old) and seeded in 24-well plates at a density of 1 × 10^5^ cells/well. After 24 h, the culture medium was replaced with GelA medium supplemented with RANKL (50 ng/mL), M-CSF (40 ng/mL), and TGF-β2 (1 ng/mL). The medium was refreshed every 2 days. After 7 days of induction, cells were fixed with 10% neutral-buffered formalin and stained with TRAP solution according to the manufacturer’s protocol to assess osteoclast formation. Cell viability was assessed using an MTT assay, and absorbance was measured with a microplate reader to calculate survival rates.

### 2.9 Tumor resection and postoperative prevention effects *in vivo*

To evaluate the antitumor effect of the hydrogel, MNNG/HOS tumor-bearing nude mice were randomly divided into four groups (n = 4 per group): Control (PBS injection), GVs@Gel (blank hydrogel with ginger vesicles), and GVs-DOX@Gel (hydrogel loaded with both GVs and doxorubicin). Due to the localized delivery format, a free DOX-only group was not included, as unencapsulated DOX rapidly diffuses and fails to mimic clinical hydrogel-mediated retention. When tumors reached ∼50 mm^3^, mice were anesthetized using isoflurane (4% induction in 100% oxygen, followed by 2% maintenance via nose cone), and 100 μL of PBS, GVs@Gel, or GVs-DOX@Gel was injected peritumorally (single administration). Body weight and tumor volume were monitored daily. On day 10, mice were euthanized by intraperitoneal injection of sodium pentobarbital (150 mg/kg) followed by cervical dislocation to ensure irreversible loss of consciousness, in accordance with AVMA euthanasia guidelines, and tumors were harvested for histological analysis. Hematoxylin and eosin (H&E), Ki67, and TUNEL staining were performed on tumor sections to investigate the therapeutic mechanism of GVs-DOX@Gel. The sample size (n = 4) was selected based on preliminary power analysis and ethical guidelines to reduce animal use while allowing detection of statistically significant therapeutic effects.

### 2.10 DOX-GVs@Gel promotes surgical wound healing *in vivo*

A full-thickness excisional wound model was established in male Sprague-Dawley rats (6–8 weeks old) to assess the wound healing potential of the hydrogels. Anesthesia was induced via intraperitoneal injection of a mixture containing ketamine (80 mg/kg) and xylazine (10 mg/kg) in 0.9% saline (1 mL/kg), ensuring surgical plane anesthesia with loss of pedal withdrawal reflex. Animals were maintained on a heating pad to preserve body temperature (37°C ± 0.5°C) throughout the procedure. Under anesthesia, rats were shaved and subjected to an 8 mm diameter circular skin excision using a biopsy punch. Animals were randomly assigned to three groups (n = 3 per group): PBS, GVs@Gel, and GVs-DOX@Gel. At predetermined time points, skin tissues from the wound sites were collected, fixed in paraffin, and subjected to histological evaluation using H&E and Masson’s trichrome staining.

### 2.11 Statistical analysis

All data are presented as mean ± standard deviation (SD). Statistical comparisons between groups were performed using two-tailed t-tests in GraphPad Prism 6. Statistical significance was indicated as ∗*p* < 0.05, ∗∗*p* < 0.01, ∗∗∗*p* < 0.001, and ∗∗∗∗*p* < 0.0001, respectively.

## 3 Results

### 3.1 Preparation and characterization of DOX-GVs loaded CMCSMA/TA hydroge

**FIGURE 2:**
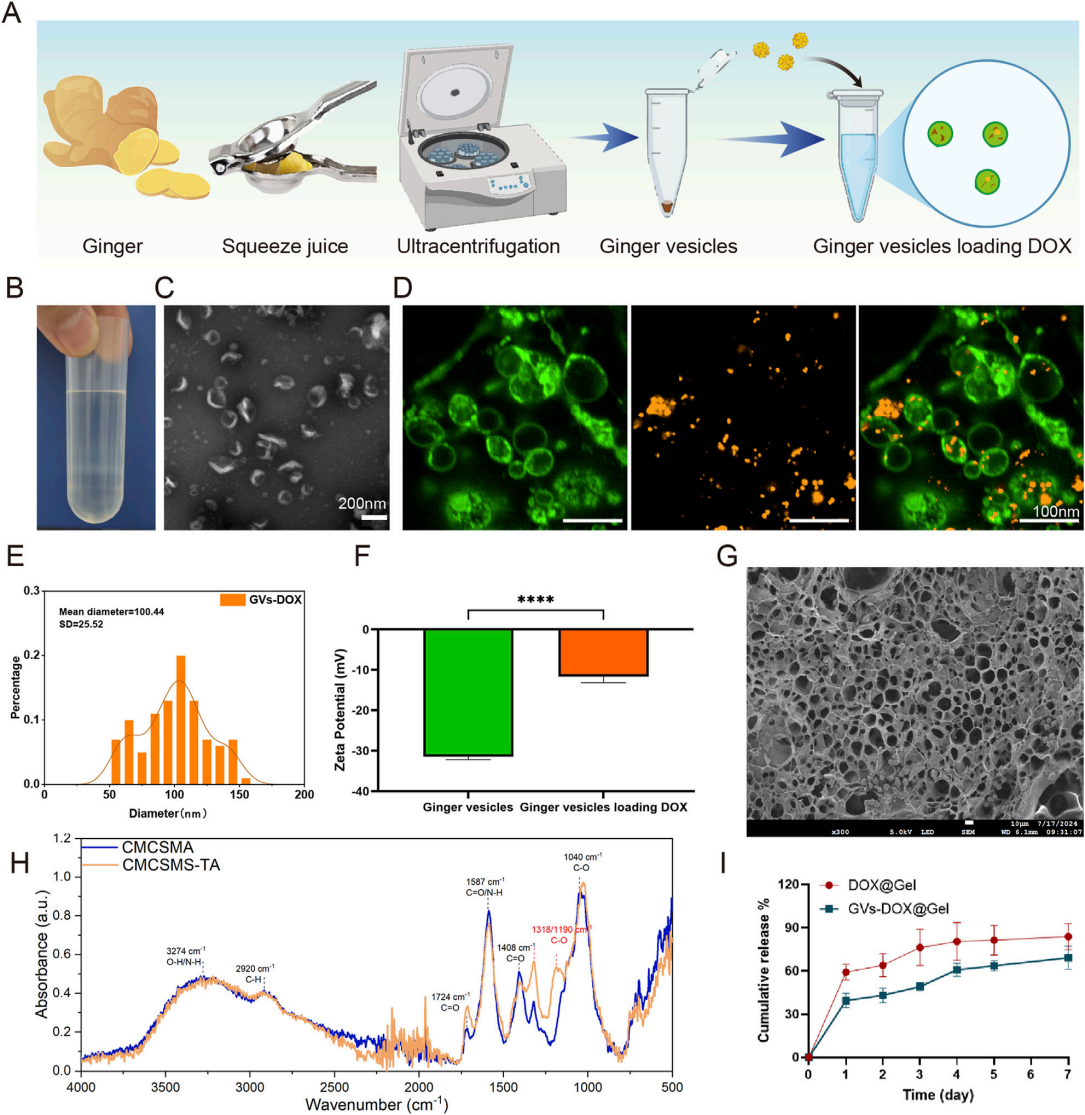
Characterization of DOX@ginger vesicles (GVs-DOX) and CMCSMA-TA hydrogel loaded with DOX@ginger vesicles (GVs-DOX@Gel). **(A)** Schematic diagram of the synthesis process of GVs-DOX. **(B)** Representing images of sucrose density gradient centrifugation. **(C)** Transmission electron microscopy (TEM) images depicting ginger vesicles. **(D)** Flurosence images shows the GVs containing DOX. **(E)** Particle size distribution of GVs-DOX. **(F)** Zeta potential of GVs and GVs-DOX. **(G)** SEM images shows the microstructure of GVs-DOX@Gel. **(H)** Fourier transform infrared (FTIR) spectra of CMCSMA and CMCSMA-TA hydrogel. **(I)** Cumulative DOX release profiles of DOX@Gel and GVs-DOX@Gel.

GVs were isolated via size exclusion chromatography and ultrafiltration (Figures 2A,B) and characterized using TEM (Figure 2C). GVs were predominantly concentrated in the fourth fraction, while negligible amounts were detected in other fractions or in unpurified samples. To confirm successful encapsulation of DOX, GVs were labeled with PKH67 (green fluorescence), and DOX was labeled with CY5 (red fluorescence). Fluorescence microscopy revealed a strong overlap of green and red signals in the merged images, confirming efficient colocalization of DOX within the GVs (Figure 2D). The hydrodynamic diameter and zeta potential of GVs-DOX were measured by dynamic light scattering (DLS). The particles exhibited an average diameter of 100.4 ± 25.52 nm and a zeta potential of −11.66 mV, indicating colloidal stability (Figures 2E,F). To characterize the CMCSMA/TA hydrogel encapsulating GVs-DOX, scanning electron microscopy was performed on lyophilized samples. The hydrogel exhibited a loose, highly porous architecture with interconnected pores ranging from 50 to 200 μm (Figure 2G), a structure favorable for nutrient diffusion, cell infiltration, and tissue regeneration. Fourier-transform infrared (FT-IR) spectroscopy was used to analyze the molecular interactions between CMCSMA and TA. Characteristic peaks of CMCSMA appeared at 1,652 cm^−1^ (amide I, C=O stretch) and 1,550 cm^−1^ (amide II, N–H bend), while TA showed a peak at 3,340 cm^−1^ (–OH stretch). In the hydrogel spectrum, CMCSMA’s amide peaks were significantly weakened, and the–OH peak shifted to 3,205 cm^−1^ with notable broadening. These changes suggest the formation of hydrogen bonds and potential ionic or covalent interactions between CMCSMA and TA, indicating successful hydrogel formation (Figure 2H). Rheological analysis revealed that the storage modulus (G′) exceeded the loss modulus (G″), confirming the formation of a stable, crosslinked hydrogel network (Supplementary figure S1). To evaluate the drug release behavior, an *in vitro* release study of DOX was conducted in PBS. After 24 h, only 39.46% of DOX was released from GVs-DOX@Gel, compared to 59.15% from DOX@Gel without GVs encapsulation (Figure 2I). This demonstrates that GVs encapsulation effectively slowed the release rate, supporting sustained drug delivery and prolonged retention at the tumor site. In summary, the CMCSMA/TA hydrogel successfully encapsulated DOX-loaded GVs, forming a stable, porous, and biocompatible structure with controlled and sustained drug release, ideal for localized tumor therapy.

### 3.2 Intraoperative hemostasis Performance of CMCSMA/TA hydrogel

**FIGURE 3:**
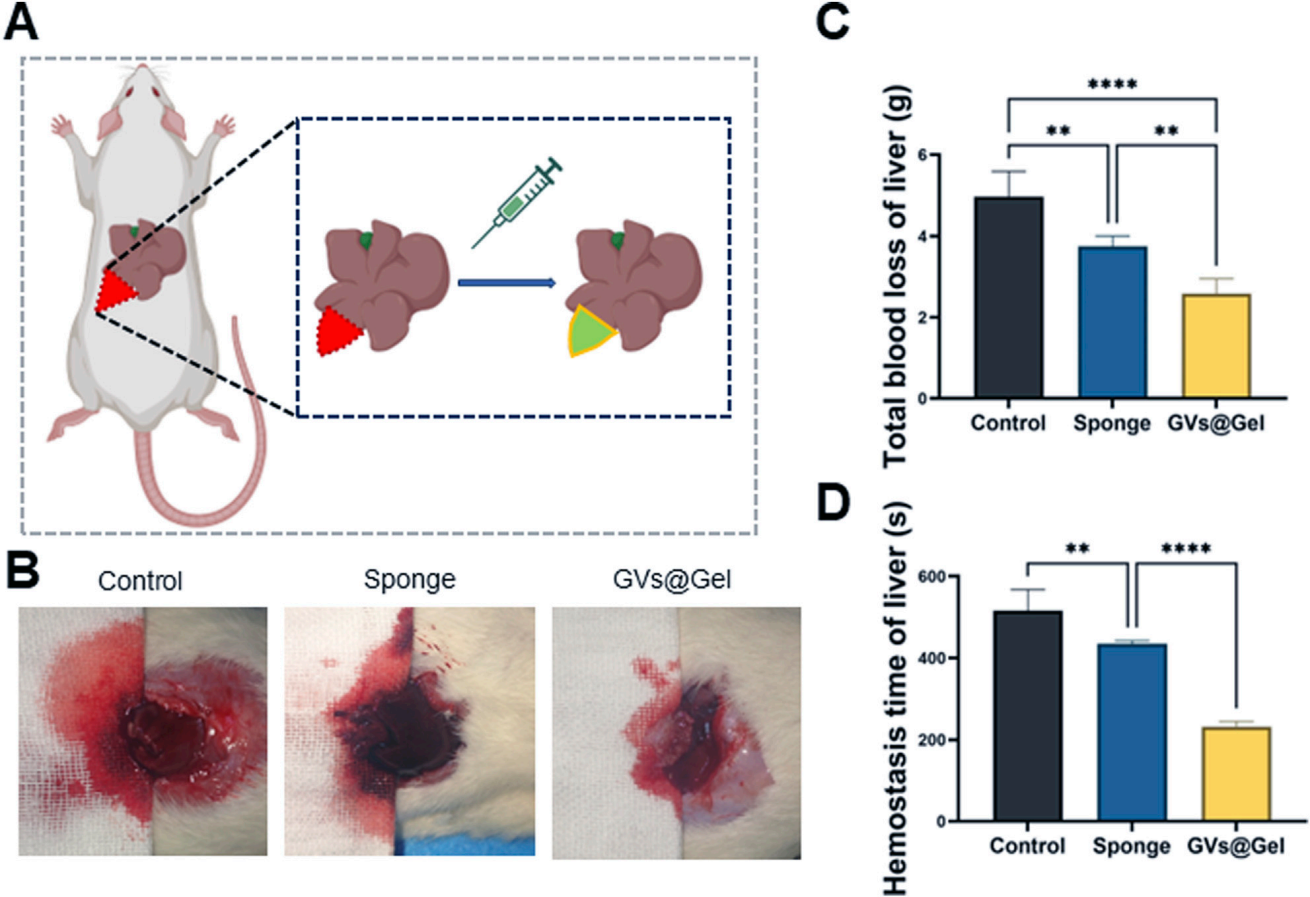
Hemostatic property of the GVs@Gel hydrogel. **(A)** Schematic diagram of the liver incision model; **(B)** photographs of blood trace; **(C)** Statistics of total blood loss; **(D)** Statistics of hemostasis time without treatment (Control) or with medial sponge and hydrogel treatments. *p < 0.05, **p < 0.01, ***p < 0.001, or ****p < 0.0001, mean ± SD, n = 3.

Severe bleeding is a leading cause of mortality, particularly in combat and emergency trauma settings. Blood transfusions after massive hemorrhage can result in complications such as coagulopathy, infection, and multi-organ failure. Therefore, rapid and effective hemostasis is critical to reducing blood loss and improving survival rates (Gao et al., 2020). To assess the hemostatic performance of the CMCSMA/TA hydrogel, we employed a rat liver incision bleeding model (Figure 3). Both the medical sponge and the CMCSMA/TA hydrogel significantly reduced blood loss compared to the untreated control. In the control group, blood loss averaged 4.97 g, which was reduced to 3.75 g with the medical sponge and further decreased to 2.59 g with the hydrogel. Similarly, hemostatic time decreased from 516 s in the control group to 434 s with the sponge and 232 s with the hydrogel. These results indicate that the CMCSMA/TA hydrogel exhibits superior hemostatic efficacy compared to the medical sponge. This enhanced hemostatic performance is likely attributable to both the intrinsic bioactivity and the adhesive properties of the hydrogel. Chitosan’s amino groups become protonated in blood, forming -NH_3_^+^ groups that attract negatively charged platelets, promoting red blood cell aggregation and thrombus formation (Yang et al., 2020). TA contributes through vasoconstrictive effects and its interaction with plasma proteins to facilitate clotting (Yang et al., 2020). Additionally, the hydrogel’s strong tissue adhesion enables it to effectively seal wound sites and block bleeding. In summary, the CMCSMA/TA hydrogel demonstrates potent hemostatic activity due to the synergistic effects of its bioactive components and adhesive capability, making it a promising candidate for intraoperative bleeding control.

### 3.3 Anti-tumor effects of DOX-GVs *in vitro*

**FIGURE 4:**
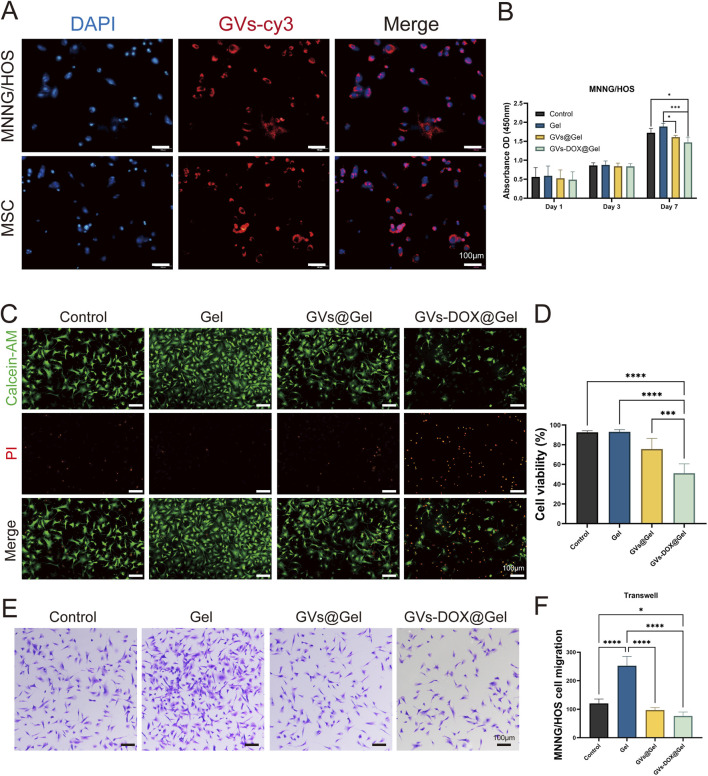
*In vitro* anticancer activity tests. **(A)** CLSM images of cellular uptake of DOX in MSC and MNNG/HOS cells. **(B)** Cell viability of MNNG/HOS cells with different treatments. **(C)** Live/dead MNNG/HOS cell viability evaluation of Control, Gel, GVs@Gel and GVs-DOX@Gel. **(D)** Quantitatively analysis of MNNG/HOS cell viability. **(E,F)** Transwell asssy exhibited the effect of Control, Gel, GVs@Gel and GVs-DOX@Gel on the migration of MNNG/HOS cell. *p < 0.05, **p < 0.01, ***p < 0.001, or ****p < 0.0001, mean ± SD, n = 3.

The cellular uptake of GVs-DOX nanoparticles (NPs) was evaluated in MNNG/HOS osteosarcoma cells and bone marrow-derived mesenchymal stem cells (BM-MSCs) using fluorescence microscopy. To visualize uptake, DOX was replaced with Cy5-labeled DOX (red fluorescence), while cell nuclei were counterstained with DAPI (blue). As shown in Figure 4A, cells treated with Cy5-DOX@GVs exhibited strong red fluorescence, indicating enhanced cellular internalization of the nanoparticles. To examine the *in vitro* therapeutic effects, MNNG/HOS cells were co-cultured with various hydrogel formulations. Compared to the Control and Gel groups, the GVs-DOX@Gel group displayed a significantly higher proportion of dead cells, as evidenced by more intense red fluorescence in live/dead staining images (Figures 4B–D). Furthermore, Transwell migration assays revealed that GVs-DOX@Gel treatment substantially inhibited tumor cell migration compared to other groups (Figures 4E,F).

### 3.4 Pro-tissue repair effects of DOX-GVs *in vitro*

**FIGURE 5:**
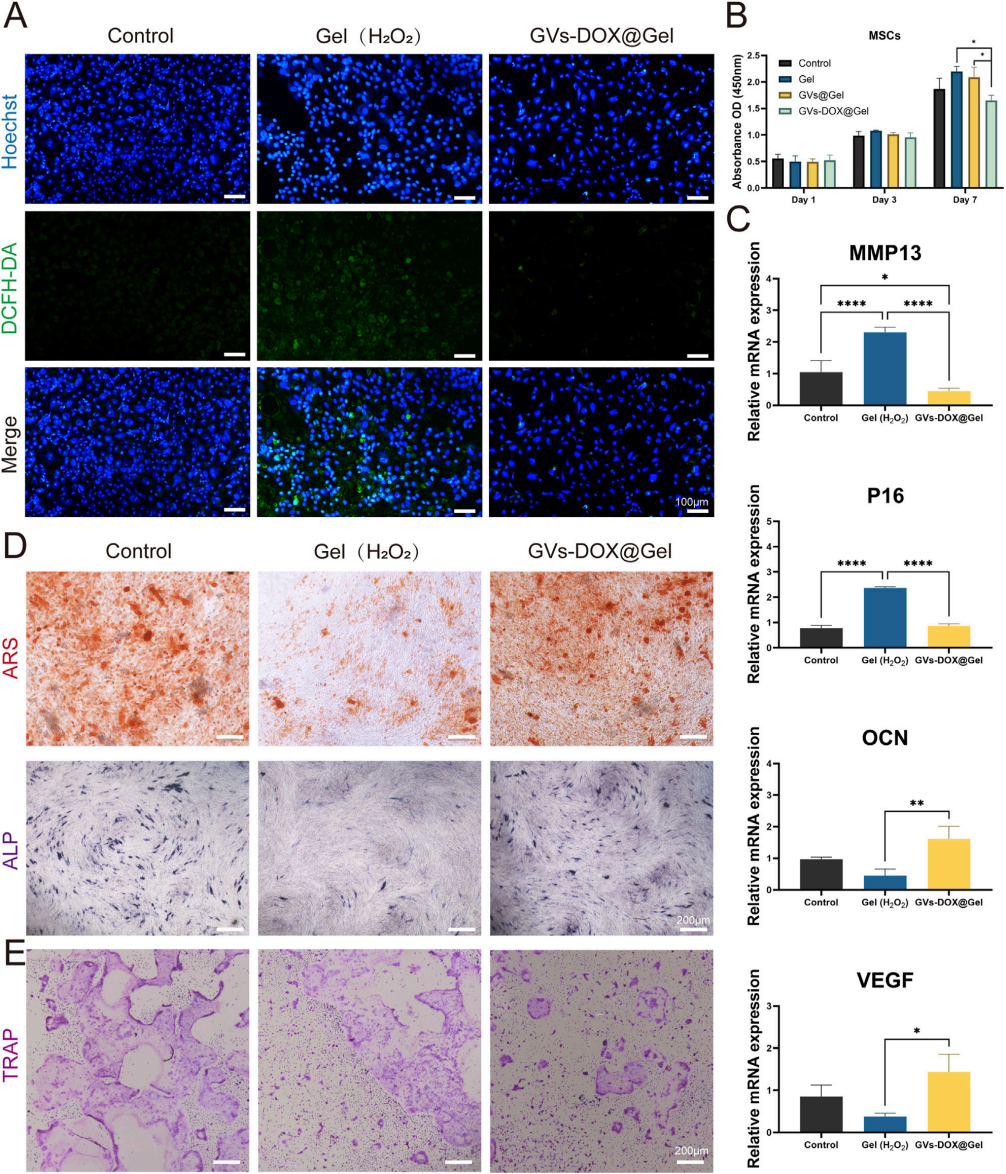
*In vitro* antioxidant and anti-inflammatory tests. **(A)** Intracellular ROS scavenging by GVs-DOX@Gel hydrogels in a H_2_O_2_-treated rat BM-MSC cells. **(B)** Cell viability of BM-MSC cells with different treatments. **(C)** Expression of inflammatory mediators MMP13, P16, OCN and VEGF in LPS-treated cells. **(D)** ARS and ALP staining of BM-MSC cells after osteoginc induction with different treatments. **(E)** Osteoclast induction evaluation representative images with different treatments. *p < 0.05, **p < 0.01, ***p < 0.001, or ****p < 0.0001, mean ± SD, n = 3.

Good biocompatibility is essential for materials intended for biomedical applications. To evaluate the biocompatibility of the GVs-DOX@Gel hydrogel, bone marrow-derived mesenchymal stem cells (BM-MSCs) were used. Intracellular ROS levels were measured using the DCFH-DA fluorescent probe. As shown in Figure 5A, H_2_O_2_ treatment markedly increased ROS generation, whereas treatment with GVs-DOX@Gel resulted in minimal green fluorescence, indicating a significant reduction in cellular ROS levels. Cytotoxicity was assessed using the CCK-8 assay (Figure 5B), which showed that the hydrogel exhibited good cell viability, confirming its biocompatibility with BM-MSCs.

To further explore the hydrogel’s therapeutic potential, its anti-inflammatory, anti-senescent, and pro-osteogenic properties were evaluated. BM-MSCs were stimulated with H_2_O_2_ or lipopolysaccharide (LPS), a potent pro-inflammatory agent. Quantitative PCR analysis of gene expression (Figure 5C) revealed that GVs-DOX@Gel treatment significantly downregulated the expression of the pro-inflammatory gene MMP13 and the senescence marker P21, while maintaining the expression of osteogenic (OCN) and angiogenic (VEGF) markers, indicating a favorable pro-regenerative profile.

The osteogenic potential of GVs-DOX@Gel was further validated using alizarin red (ARS) and alkaline phosphatase (ALP) staining in MC3T3-E1 cells. As shown in Figure 5D, treatment with GVs-DOX@Gel significantly enhanced mineralization and ALP activity compared to the control, suggesting promotion of osteogenic differentiation. Additionally, tartrate-resistant acid phosphatase (TRAP) staining (Figure 5E) showed a notable reduction in TRAP-positive cells following treatment, indicating inhibition of osteoclastic differentiation in BMDMs. MTT assays further confirmed that GVs-DOX@Gel suppressed osteoclast proliferation and activity. Collectively, these findings demonstrate that GVs-DOX@Gel possesses excellent biocompatibility and exhibits multifunctional properties, including antioxidant, anti-inflammatory, anti-senescent, osteogenic, and anti-osteoclastic effects, making it a promising candidate for postoperative bone regeneration and osteosarcoma therapy.

### 3.5 Tumor resection and postoperative prevention effects *in vivo*

**FIGURE 6:**
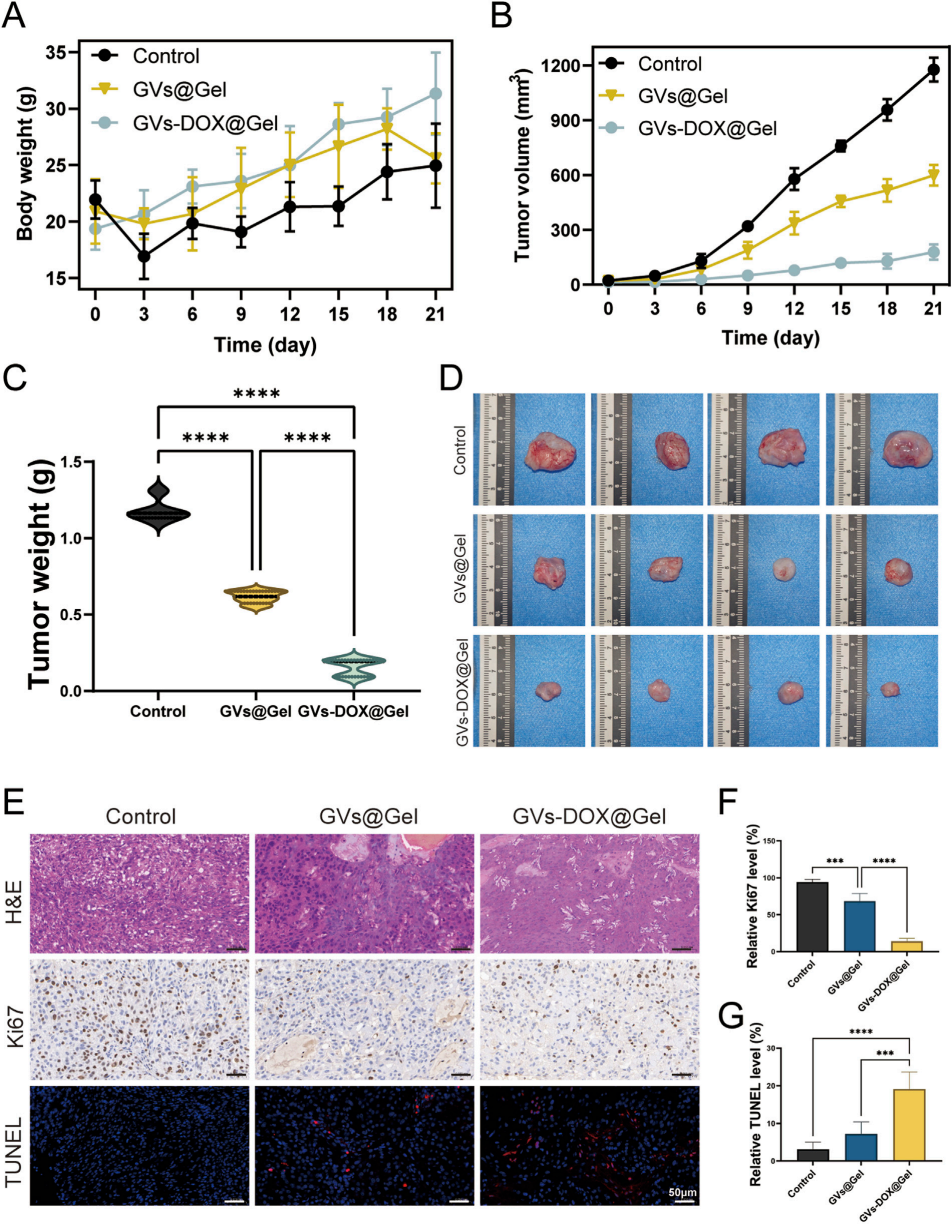
*In vivo* anticancer effect to MNNG/HOS bearing balb/c mice (n = 4). **(A)** Body weight of MNNG/HOS bearing balb/c mice as a function of time after different treatments. **(B)** Tumor volume of MNNG/HOS bearing balb/c mice as a function of time after different treatments. **(C)** Tumor weights of MNNG/HOS tumor-bearing mice with various treatments. **(D)** Photographs of the excised tumors. **(E)** The staining of H&E, Ki67, and TUNEL of tumor slices. Quantative analysis of relative Ki67 **(F)** and **(G)** TUNEL level of tumor slices.

To further evaluate the *in vivo* antitumor efficacy of the GVs-DOX@Gel hydrogel, we established an osteosarcoma xenograft model by subcutaneously injecting MNNG/HOS cells into Balb/c nude mice. Once tumor volumes reached approximately 50 mm^3^, mice received peri-tumoral injections of PBS (control), GVs@Gel, or GVs-DOX@Gel. Body weight and tumor volume were monitored daily for 21 days. As shown in Figures 6A,B, the GVs-DOX@Gel group exhibited significant tumor growth inhibition compared to both the PBS and GVs@Gel groups. Although the GVs@Gel group initially showed some suppression of tumor growth, tumors began to grow rapidly after day 6. In contrast, GVs-DOX@Gel maintained continuous tumor suppression throughout the observation period (Figures 6C,D). This sustained effect can be attributed to the hydrogel’s role as a localized drug reservoir, enabling gradual DOX release and maintaining therapeutic drug concentrations at the tumor site.

Histological analysis of excised tumors further confirmed the superior antitumor activity of GVs-DOX@Gel. H&E staining, Ki67 immunohistochemistry, and TUNEL fluorescence staining were conducted. Ki67 staining revealed significantly reduced tumor cell proliferation in the GVs-DOX@Gel group, while TUNEL staining showed markedly increased apoptosis, as indicated by stronger red fluorescence compared to the other groups (Figures 6E-G). Together, these results demonstrate that GVs-DOX@Gel hydrogel effectively inhibits tumor proliferation and promotes apoptosis *in vivo* by providing sustained DOX release, confirming its strong potential for postoperative osteosarcoma treatment.

### 3.6 DOX-GVs@Gel promotes surgical wound healing *in vivo*

**FIGURE 7:**
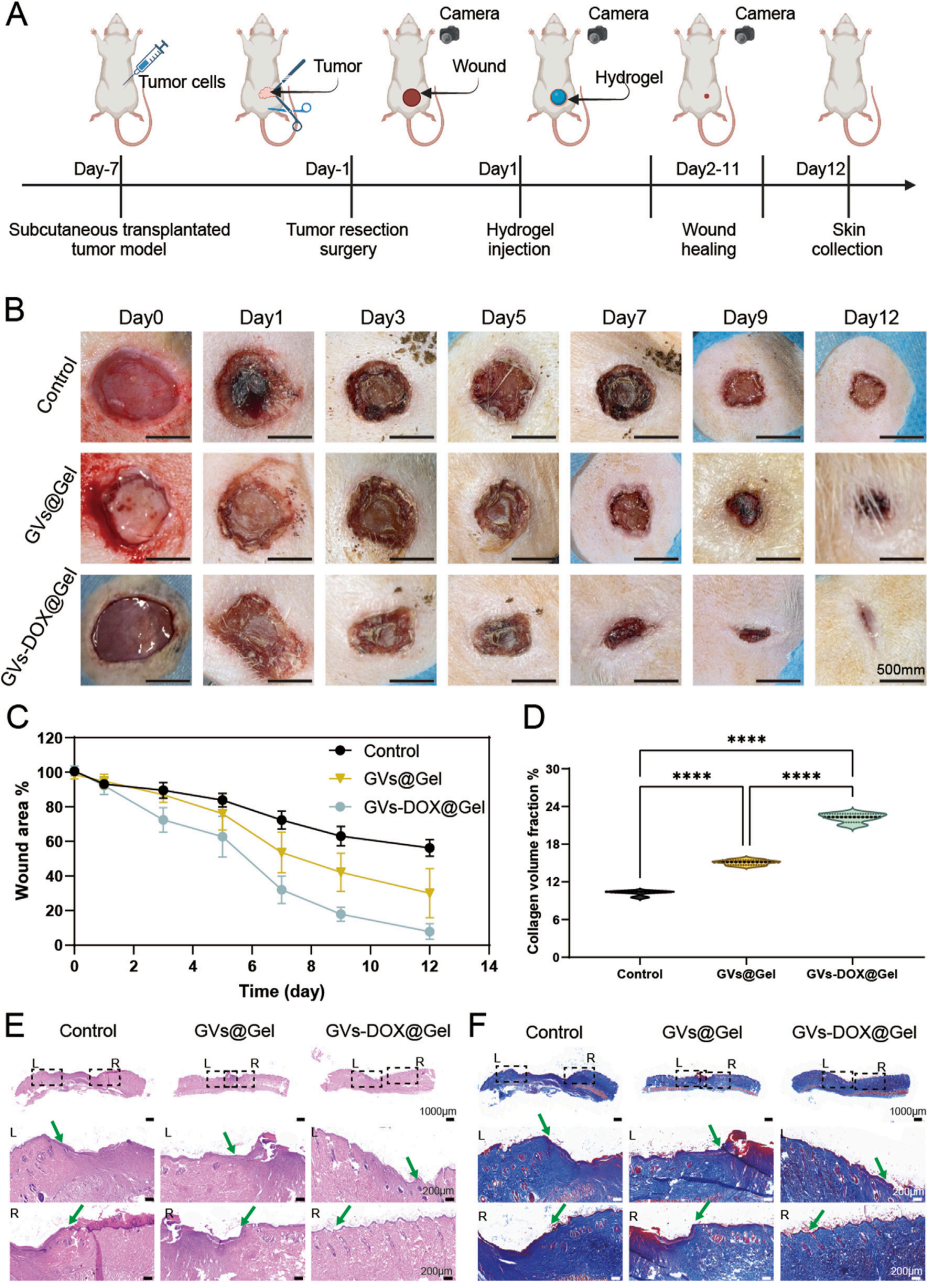
Wound healing ability. **(A)** Schematic diagram of postoperative trauma model of osteosarcoma and wound healing with different treatment. **(B)** Photography of representative wounds at different time after different treatments (scale bar: 5 mm). **(C)** Quantitative evaluation of wound healing ratio as a function of time after treatment. H&E **(E)** and Massion **(D,F)** staining of healing wounds from rat 12 days received different treatments, respectively (scale bar: 100 μm).

Surgical resection remains a key treatment for osteosarcoma, yet challenges such as local tumor recurrence and delayed wound healing persist. To evaluate the *in vivo* therapeutic potential of GVs-DOX@Gel in addressing these issues, we established a full-thickness excisional skin wound model in rats to simulate postoperative healing. Rats were divided into three groups: Control (treated with PBS), Gel (treated with CMCSMA/TA hydrogel), and GVs-DOX@Gel. First, as shown in Figure 7A, a subcutaneous osteosarcoma xenograft model was created by injecting MNNG/HOS cells into immunodeficient rats. Seven days later, tumors were surgically resected to simulate postoperative trauma. Following resection, the wounds were treated with the respective formulations. As shown in Figure 7B, the GVs-DOX@Gel group exhibited significantly faster and more complete wound closure compared to both the Control and Gel groups. Quantitative analysis revealed that the GVs-DOX@Gel group consistently showed the smallest wound area on days 3, 7, and 12 post-treatment (Figures 7C,D). To further assess tissue regeneration, skin samples collected on day 14 were analyzed using H&E and Masson staining. H&E staining (Figure 7E) revealed that the GVs-DOX@Gel group had a thicker regenerated epidermis (green arrow) and more skin appendages, such as glands and hair follicles. Masson staining (Figure 7F) demonstrated enhanced collagen deposition in the GVs-DOX@Gel group, indicating improved dermal remodeling. Notably, both the Gel and GVs-DOX@Gel groups outperformed the Control group in promoting wound healing, suggesting that the CMCSMA/TA hydrogel contributes to wound sealing and tissue repair. However, the superior outcomes observed in the GVs-DOX@Gel group highlight the added therapeutic benefits of GVs-DOX, particularly in enhancing fibroblast activity and collagen synthesis. These results demonstrate that GVs-DOX@Gel not only supports effective postoperative tumor therapy but also significantly accelerates wound healing, making it a promising candidate for integrated osteosarcoma treatment.

## 4 Discussion

Effective postoperative management of osteosarcoma demands strategies that simultaneously prevent tumor recurrence and promote tissue regeneration. Injectable hydrogels have emerged as multifunctional therapeutic platforms that serve as both temporary scaffolds for residual tumor ablation and sustained matrices for regenerative healing (Zhang et al., 2024; Chen et al., 2024). Their tunable mechanical properties, bioactive ligand presentation, and controlled drug release enable precise modulation of the physicochemical microenvironment, thereby directing key cellular processes such as proliferation, differentiation, and matrix remodeling (Chen et al., 2024; Liu et al., 2021; Tallapaneni et al., 2022). Redox-responsive hydrogels, in particular, exhibit enhanced therapeutic potential by scavenging ROS, modulating macrophage polarization toward the M2 phenotype, and promoting angiogenesis and collagen deposition (Wang et al., 2020). Recent advances have led to the development of hydrogels with multimodal therapeutic capabilities, including chemo-photothermal synergy, pH/NIR-triggered release, and antibacterial properties. However, challenges remain in engineering hydrogels that can continuously neutralize ROS while maintaining structural stability for staged tissue repair.

In this study, we developed a novel multifunctional adhesive hydrogel to address the complex postoperative challenges faced by elderly osteosarcoma patients—namely, tumor recurrence, inadequate hemostasis, chronic inflammation, and impaired regeneration. Composed of CMCSMA and TA, the hydrogel forms a dynamically crosslinked network with excellent tissue adhesion, adaptability to moist wound environments, and rapid hemostasis. Incorporation of GVs loaded with DOX further enhances the system by enabling dual therapeutic functions: localized chemotherapy and tissue repair through antioxidative and anti-inflammatory actions, as well as osteogenic promotion.

The translation of hydrogel-based medical products into clinical applications necessitates navigating complex regulatory frameworks to ensure safety, efficacy, and quality. For hydrogels composed of natural ingredients like chitosan and tannic acid, regulatory approval pathways often prioritize biocompatibility, biodegradability, and toxicity profiling, as these materials are generally considered safer than synthetic alternatives (Capella-Monsonís et al., 2024; Kolipaka et al., 2024). However, the incorporation of active pharmaceutical ingredients (e.g., DOX-loaded GVs) introduces additional regulatory scrutiny, requiring rigorous evaluation of drug release kinetics, localized therapeutic efficacy, and long-term safety (Khosravimelal et al., 2021). Compliance with Good Manufacturing Practices (GMP) is critical for scalability, particularly for dynamically crosslinked networks that demand precise control over synthesis parameters to ensure batch-to-batch consistency. While natural polymers like chitosan may benefit from existing regulatory precedents in wound care and tissue engineering, hybrid systems combining drug delivery and tissue repair functions must address multifunctional regulatory criteria, including combination product guidelines (Capella-Monsonís et al., 2024; Lu et al., 2024).

The scalability of hydrogel manufacturing relies heavily on the compatibility of raw materials with industrial processes. Natural ingredients such as ginger extract, chitosan, and tannic acid offer advantages in cost-effective sourcing and compatibility with GMP-grade synthesis, as highlighted by the feasibility of multilayered device production using similar polymers (Baek et al., 2023; Patel et al., 2024). The dynamic crosslinking between CMCSMA and TA simplifies fabrication by enabling self-assembly under physiological conditions, reducing reliance on energy-intensive crosslinking methods (Cao et al., 2021; Yin et al., 2023). However, challenges persist in scaling up complex systems like DOX-loaded GVs, which require stringent control over encapsulation efficiency and sterilization protocols to maintain therapeutic functionality. Advances in modular manufacturing platforms, such as 3D bioprinting and microfluidic encapsulation, could enhance reproducibility for hydrogels designed for moist wound environments, though these technologies must balance precision with cost constraints (Baniasadi et al., 2024).

The cost-effectiveness of hydrogel-based therapies is influenced by material availability, manufacturing complexity, and therapeutic outcomes. Natural polymers like chitosan and tannic acid are inherently low-cost and abundant, reducing raw material expenses compared to synthetic alternatives. The dual therapeutic functionality of DOX-loaded GVs—combining chemotherapy and tissue repair—may offset costs by minimizing the need for adjunct treatments, as demonstrated in preclinical models of localized drug delivery (Baek et al., 2023). The dual therapeutic functionality of DOX-loaded GVs—combining chemotherapy and tissue repair—may offset costs by minimizing the need for adjunct treatments, as demonstrated in preclinical models of localized drug delivery (Cao et al., 2021). However, the economic viability of GMP-compliant production depends on optimizing energy-efficient processes, particularly for dynamically crosslinked networks requiring precise temperature and pH control during synthesis (Zhao et al., 2022). Long-term cost savings could arise from reduced hospitalization times due to the hydrogel’s rapid hemostasis and anti-inflammatory properties, though real-world implementation must address reimbursement frameworks and healthcare infrastructure limitations.

To further support translational feasibility, we have also considered the scalability, sterilization compatibility, and long-term storage of the hydrogel system. GV extraction was performed using well-established ultracentrifugation protocols, and we observed consistent particle size and zeta potential across batches (as shown in DLS and TEM results), suggesting good reproducibility. The hydrogel components—CMCSMA and TA—are derived from widely available natural materials, and their self-assembly at physiological conditions simplifies fabrication and reduces energy input, improving compatibility with GMP-scale production. Regarding sterilization, chitosan-based hydrogels have been shown in literature to withstand γ-ray irradiation without significant degradation in mechanical or adhesive properties (Wu et al., 2024); our formulation, composed of polyphenol-crosslinked networks, is expected to retain stability under similar conditions, though detailed analysis will be explored in future work. For storage, both GVs and hydrogels demonstrated stable properties when maintained at 4°C for several weeks, consistent with previous reports on plant-derived vesicle and hydrogel preservation. Together, these features highlight the material’s manufacturing feasibility, shelf stability, and potential for clinical translation. Lifecycle assessments comparing hydrogel-based therapies to conventional treatments (e.g., systemic chemotherapy or standard wound dressings) will be essential to validate cost-benefit ratios.

Hemostasis is particularly critical in geriatric osteosarcoma patients, who are more susceptible to hemorrhagic complications (Shakouripour et al., 2025; Zhang K. et al., 2023). Our hydrogel achieved a 55% reduction in bleeding time in a rat liver hemorrhage model, outperforming traditional gauze. This hemostatic effect is achieved through: (1) rapid tissue adhesion via catechol-metal coordination chemistry, and (2) activation of coagulation cascades through chitosan’s cationic charge and tannic acid’s polyphenolic composition (Guo et al., 2022; Deng et al., 2022). The fast response (<4 min) reduces intraoperative blood loss (p < 0.01 vs control) and potentially prevents secondary hemorrhagic events by maintaining local hemostatic stability.

A key innovation of this system lies in the use of GVs as multifunctional nanocarriers. These vesicles encapsulate hydrophobic antioxidants—such as curcumin, gingerols, and shogaols—with synergistic ROS-scavenging and anti-inflammatory properties (Huang et al., 2024; Zeng et al., 2024). Simultaneously, they mediate localized DOX delivery, thereby overcoming the limitations of systemic chemotherapy, including off-target toxicity and dose-limiting side effects (Zhang L. et al., 2023). Our findings confirm that GVs-Cy3 effectively internalizes into both MNNG/HOS osteosarcoma cells and MSCs, significantly inhibiting tumor cell proliferation and migration. This dual-targeting mechanism ensures: (1) sustained DOX release within the tumor microenvironment, enhancing local therapeutic concentration, and (2) minimized systemic toxicity. Consequently, this hydrogel maximizes intratumoral drug bioavailability and effectively eradicates residual tumor cells, addressing a major cause of recurrence and poor prognosis in osteosarcoma.

Ginger-derived vesicles (GVs), particularly ginger-derived extracellular vesicles (GDEVs) or exosome-like nanoparticles (G-ELNs), exhibit notable low immunogenicity and anti-inflammatory properties, making them promising candidates for therapeutic applications. GDEVs are characterized by their natural origin, which confers low immunogenicity due to reduced risk of immune clearance and compatibility with mammalian systems, as highlighted by their ability to prolong blood circulation without triggering immune responses *in vivo* (Han et al., 2025; Wang et al., 2025). Additionally, GVs derived from ginger (GDNVs) have been shown to eliminate pathogenic oral commensal bacteria (e.g., *Streptococcus*), which are implicated in chemoresistance and inflammation in oral cancer, further underscoring their role in mitigating infection-driven inflammation. The anti-inflammatory effects are also linked to their ability to modulate immune cell responses, such as reducing macrophage polarization toward pro-inflammatory phenotypes and enhancing tissue repair processes (Han et al., 2025).

*In vitro* analyses demonstrated the hydrogel’s potent ROS-scavenging capacity, mitigating oxidative stress—a key barrier to wound healing and osteogenesis (Zhang et al., 2024; Hou et al., 2025). The hydrogel supports redox homeostasis, reduces inflammatory markers (e.g., >60% reduction in MMP13), suppresses cellular senescence, and promotes proliferation. These actions collectively accelerate angiogenesis (evidenced by VEGF upregulation) and osteogenesis (increased ALP activity and 3.1-fold RUNX2 expression). Notably, the GVs within the hydrogel also directly induce MSC differentiation, suggesting potential to enhance bone matrix deposition and repair at the surgical site (Bargavi et al., 2024). These regenerative effects align with known mechanisms of plant-derived extracellular vesicles (PDEVs), which carry growth factors and modulate matrix metalloproteinases, making them highly suitable for osteoinductive applications—especially in elderly patients with diminished bone regenerative capacity.

*In vivo* studies using a rat osteosarcoma resection model further validated the hydrogel’s multimodal therapeutic performance. Compared to GVs@Gel treatment, our engineered hydrogel showed superior efficacy in three key areas: (1) rapid hemostasis via optimized tissue adhesion, (2) effective tumor suppression through sustained DOX release, and (3) enhanced wound regeneration driven by antioxidative and anti-inflammatory modulation. These results confirm that the hydrogel simultaneously coordinates hemostasis, redox balance, inflammation control, and localized chemotherapy—addressing multiple dimensions of postoperative osteosarcoma care.

While the current findings strongly support the hydrogel’s clinical potential, several limitations warrant consideration. First, all *in vivo* studies were conducted in small animals, and future validation in large animal models is essential to better replicate human skeletal and biomechanical conditions. Second, the aged immune microenvironment—marked by immunosenescence and chronic inflammation—may influence therapeutic outcomes; thus, aged or immunocompromised models should be employed to evaluate immune compatibility. Additionally, further optimization of drug-loading efficiency, long-term degradation behavior, and integration with complementary therapies such as photothermal modulation or immune activation will enhance the system’s translational potential.

## 5 Conclusion

In summary, we developed an injectable adhesive hydrogel system encapsulating DOX and GVs, demonstrating strong localized antitumor activity and enhanced wound healing capabilities. By enabling targeted DOX release at the tumor site, the hydrogel improves chemotherapeutic efficacy while minimizing systemic toxicity. Simultaneously, the antioxidant and anti-inflammatory properties of GVs create a regenerative microenvironment that promotes tissue repair and mitigates postoperative inflammation. Together, these features address critical clinical challenges of tumor recurrence and impaired wound healing after surgical resection. This multifunctional hydrogel not only serves as a promising postoperative adjuvant therapy but also offers a versatile platform for integrating diverse bioactive agents in broader biomedical applications.

## Data Availability

The original contributions presented in the study are included in the article/Supplementary Material, further inquiries can be directed to the corresponding author.
