# Supplementary material for: Dual-functional injectable adhesive hydrogel delivering ginger-derived doxorubicin vesicles for osteosarcoma recurrence suppression and post-resection wound healing
Source: Front Bioeng Biotechnol. 2025 Jun 19;13:1609673. doi: 10.3389/fbioe.2025.1609673 (PMC12223425; doi:10.3389/fbioe.2025.1609673)
Supplement: Supplementary file 1 [file Datasheet1.docx]

Supplementary Information for

## Dual-Functional Injectable Adhesive Hydrogel Delivering Ginger-Derived Doxorubicin Vesicles for Osteosarcoma Recurrence Suppression and Post-Resection Wound Healing

## Supplementary Materials and Methods

**1. Cell and Animal**

Human MNNG/HOS osteosarcoma cell (MNNG/HOS) and osteoblast cell line (MC 3T3-E1) were obtained from the Chinese Academy of Science cells Bank (Shanghai, China) and maintained in Dulbecco’s modified Eagle medium (DMEM) with 10% FBS and 1% penicillin−streptomycin. Sprague-Dawley rats (80 g, female) were sacrificed by cervical dislocation, and femurs and tibias were separated from hind limbs. The ends of the femur or tibia were removed, and the bone marrow was flushed out with 1 ml DMEM/F12 medium. The bone marrow was repeatedly washed to generate a single-cell suspension that was centrifuged at 1000 rpm for 5 min. The supernatant was removed, and cells were washed with DMEM/F12 and centrifuged for an additional 5 min. Finally, the supernatant was removed, and cells were resuspended in DMEM/F12 medium containing 10% fetal bovine serum (FBS) and 1% penicillin-streptomycin. Cells isolated from one hind limb were plated in a 25-cm2 dish and incubated at 37 °C with 5% CO2, which was defined as passage 0 (P0). After 24 h, cells were washed with PBS twice to remove non-adherent cells. When cell confluency was greater than 90%, the cells were secondarily cultured, and the passage number was increased by one. Cells from passages 2 and 3 were used to further studies. The nude female mice, 20−22 g, were purchased from SPF (Beijing) Biotechnology Co., Ltd. All animal experiments were carried out in accordance with guidelines evaluated and approved by the ethics committee of Tianjin tumor hospital.

**2. Synthesis of carboxymethyl chitosan methacryloyl (CMCSMA)**

Chitosan (2.0 g) was immersed in 25 mL of 50 wt % NaOH solution for 24 hours at room temperature to achieve swelling and alkalization. The alkalized polymer was subsequently filtered under suction to remove excess NaOH, yielding a moist cake. Monochloroacetic acid (1.0–5.0 g, adjusted to prepare carboxymethylated derivatives with varying substitution degrees, denoted as CMCS 1–5) dissolved in 25 mL isopropanol was added dropwise to the alkalized chitosan over 20 minutes. The mixture was stirred for 8 hours at room temperature to complete the carboxymethylation reaction. Post-reaction, the slurry was filtered to remove the solvent, and the solid was dissolved in 100 mL deionized water. The solution was neutralized to pH 7.0 by adding 2.5 M HCl, followed by centrifugation (4000 rpm, 15 minutes) to remove insoluble residues. The supernatant was mixed with 150 mL ethanol to precipitate the carboxymethyl chitosan (CMCS), which was then filtered, rinsed repeatedly with ethanol, and vacuum-dried at room temperature. For methacrylation, CMCS (1.0 g) was dissolved in 100 mL deionized water, followed by sequential addition of triethylamine (5.0 mL, as a catalyst), glycidyl methacrylate (15.0 mL), and tetrabutyl ammonium bromide (5.0 g, as a phase-transfer catalyst). The reaction mixture was stirred for 3 days at ambient temperature under light protection, then heated at 60°C for 1 hour to enhance conjugation efficiency. The product was precipitated in ethanol, collected via centrifugation, and subjected to three cycles of dissolution-precipitation (water/ethanol) to eliminate unreacted monomers. Finally, the methacrylated CMCS (CMCSMA) was lyophilized to obtain a dry powder. The successful introduction of methacrylate groups was confirmed by ¹H-NMR spectroscopy.

**Table S1**. Primer sequences used for the in vivo RT–qPCR analysis of inflammatory genes.

| Gene | Forward Primers | Reverse Primers |
| --- | --- | --- |
| MMP13 | GCCCCTTCCCTATGGTGATGATG | CCGCAAGAGTCACAGGATGGTAG |
| P16 | CACCAAACGCCCCGAACAC | GCACCATAGGAGAGCAGGAGAG |
| OCN | CAGTAAGGTGGTGAATAGACTCCG | GGTGCCATAGATGCGCTTG |
| VEGF | CCACAGGGGTCCTGGCAAAG | AGCCACTCACACACACAGCC |
| GAPDH | CAAGTTCAACGGCACAGTCAAGG | ACATACTCAGCACCAGCATCACC |

**FigureS1.** Rheology analysis of CMCSMA and CMCSMA/TA hydrogels.
